# Factors Likely to Affect the Long-term Results of Ventricular Stimulation After Myocardial Infarction

**Published:** 2010-04-01

**Authors:** Beatrice Brembilla-Perrot, Pierre Yves Zinzius, Laurent Groben, Luc Freysz, Lucian Muresan, Jerome Schwartz, Raphael P Martins, Soumaya Jarmouni, Ibrahim Nossier, Nicolas Sadoul, Hugues Blangy, Arnaud Terrier De La Chaise, Pierre Louis, Olivier Selton, Daniel Beurrier, Jean Marc Sellal

**Affiliations:** Department of cardiology, CHU of Brabois, Vandoeuvre, France

**Keywords:** programmed ventricular stimulation, myocardial infarction, follow-up, reproducibility

## Abstract

**Background:**

The results of programmed ventricular stimulation (PVS) may change after myocardial infarction (MI). The objective was to study the factors that could predict the results of a second PVS.

**Methods:**

Left ventricular ejection fraction (LVEF) and QRS duration were determined and PVS performed within 3 to 14 years of one another (mean 7.5±5) in 50 patients studied systematically between 1 and 3 months after acute MI.

**Results:**

QRS duration increased from 120±23 ms to 132±29 (p 0.04). LVEF did not decrease significantly (36±12 % vs 37±13 %). Ventricular tachycardia with cycle length (CL) > 220ms (VT) was induced in 11 patients at PVS 1, who had inducible VT with a CL > 220 ms (8) or < 220 ms (ventricular flutter, VFl) (3) at PVS 2. VFl or fibrillation (VF) was induced in 14 patients at PVS 1 and remained inducible in 5; 5 patients had inducible VT and 4 had a negative 2nd PVS. 2. 25 patients had initially negative PVS; 7 had secondarily inducible VT, 4 a VFl/VF, 14 a negative PVS. Changes of PVS were related to initially increasing QRS duration and secondarily changes in LVEF and revascularization but not to the number of extrastimuli required to induce VFl.

**Conclusions:**

In patients without induced VT at first study, changes of PVS are possible during the life. Patients with initially long QRS duration and those who developed decreased LVEF are more at risk to have inducible monomorphic VT at 2nd study, than other patients.

##  Introduction

Programmed ventricular stimulation (PVS) has been reported as a valuable approach for the risk stratification [1-4]. After acute myocardial infarction (MI), the induction of a sustained ventricular tachycardia (VT) was correlated with a high risk of occurrence of ventricular arrhythmias [5-7]. The method has loss of interest after MADIT II study [8]. Low left ventricular ejection fraction (LVEF) less than 30 % alone was used as the indication for implantable defibrillator (ICD) with a significant decrease of sudden deaths in patients receiving ICD; in this last study, PVS lacks sensitivity for the prediction of the risk of ventricular arrhythmias [9]: ventricular fibrillation (VF) was more frequent in patients with a negative study than in patients with inducible ventricular arrhythmias. But, this study was also the single one that reported the lack of use of PVS for the arrhythmic risk stratification. The results differed from several older studies, that reported the use of PVS for the risk stratification [10-14]; a recent study of Daubert et al [15] in a population with low LVEF and non ischemic cardiomyopathy reported good correlation between the induction of a VT and the risk of events at ICD recording.

The purposes of the study were to look for the factors associated with the changes of PVS in patients with history of MI, without spontaneous VT at initial study and to understand why the long term follow-up could differ from initial study.

## Methods

### Study Population

The study group consisted of 50 patients recruited before 2000, with history of MI, 11 women, 39 men aged 24 to 68 years (mean 54±13) at the time of recruitment, who underwent 2 PVS. These patients were treated with ACE inhibitors and 45 of them received beta-blockers which were stopped just before electrophysiological study.

At the first study, the patients had been admitted for Q-wave acute MI and a PVS was performed at least 3 weeks after MI. Second study was performed for dizziness without syncope (n=4) or for ventricular premature beats in couplets or triplets (grade III, IVa, IVb of Lown) and decreased LVEF between 30 and 45 % among remaining patients to discuss ICD implantation.

The patients were drawn from a group of 779 consecutive patients admitted after MI for the risk stratification after MI [7]. Among these 779 patients, 204 had inducible ventricular flutter or fibrillation, 173 had inducible monomorphic VT and 402 patients had a negative study. The follow-up of most of these patients was previously reported [7,14].

The criteria for inclusion in the initial study were: 1) stable clinical conditions; 2) absence of indication of amiodarone therapy; 3) absence of associated organic disease with bad prognostic value.

### Study protocol

Non invasive and invasive studies were repeated within 3 to 14 years of one another (mean 7.5±5 years) after giving informed consent. The protocol was approved by the institutional review board.

Non-invasive studies included the recording of surface ECG and the recording of signal-averaged ECG (SAECG).  Signal averaged ECG was recorded with the Cardionics system using a frequency band of 40 to 250 Hz and was used to determine the total QRS duration. LVEF was determined by echocardiographic study (2D echo).

 Invasive studies included coronary angiography and electrophysiological study. They underwent the studies in the absence of cardioactive drugs, discontinued for at least four half-lives before testing. Kaliemia was verified and was normal. The electrophysiological tests were performed in the non sedated state.

Our protocol was previously reported [7,14]: an identical stimulation protocol was used for both studies. Right ventricular pacing was conducted at rates from 90 up to 200 b/min. Then, during spontaneous rhythm and ventricular pacing for 6 beats at two fixed cycle lengths (600 and 400 ms), a single and then a second extrastimulus were applied. If a sustained VT was not induced, the PVS was repeated from the right ventricular outflow tract. If this protocol remained negative, a third extrastimulus was added and PVS repeated from the right ventricular outflow tract and then from the right ventricular apex. The endpoint was the induction of a sustained VT or the completion of the complete PVS.

### Definitions

*Negative study:* no ventricular beats, or up to 4 repetitive ventricular responses, non-sustained VT (5 or more ventricular beats, well-tolerated and spontaneously stopping without intervention). 

*Sustained VT:* monomorphic VT, lasting more than 30 seconds or requiring therapeutic intervention.

*VF:* completely disorganized cardiac electrical activity requiring cardioversion.

*Ventricular flutter (VFl):* rapid monomorphic VT with a cycle length of < 220 ms and no isoelectrical interval between consecutive QRS complexes, generally requiring a cardioversion to stop it.

### Statistical analysis

Continuous data are presented as mean values ± standard duration. Univariate comparisons were made by the paired t-test or chi-square analysis when appropriate. A probability (p) value of < 0.05 was considered to be statistically significant.

## Results

The data of the study population at initial study are reported in Table 1. The general changes of clinical and electrophysiological data are reported in Table 2.

### Non-invasive studies

LVEF did not change significantly. Mean QRS duration on the signal-averaged ECG increased significantly on study 2 (p 0.04).

### Invasive studies

Six patients had significant coronary stenoses, cause of ischaemia at thallium exercise test and coronary angioplasty was performed before the second PVS.

Sustained monomorphic VT was induced in 11 patients on study 1; all of them had inducible VT with a cycle length of > 220 ms (n= 8) or of < 220 ms (n=3) on study 2. The rate of the inducible VT was different (> 15%) in 6 patients. However the mean heart rate of the VTs induced at the first and second study was not statistically different (p 0.06). There was a trend toward a slower rate of induced VT at the second study, from 205±35 beats/min to 190±55 b/min: the rate of inducible VT was slower at the second study, except in 2 patients who had inducible VFl at the second study. The mode of induction was different in 3 of the 11 patients with inducible VT. Three patients of this group had a revascularization with no changes at second study. The morphology of the VT differed at the first and second study in 6 patients.

 VFl (n= 13) or VF (n=1) was induced in 14 patients on study 1. Ventricular flutter was induced by 2 extrastimuli in 6 patients and 3 extrastimuli in 7 patients. Ventricular flutter remained inducible in 4 patients; 5 patients had inducible VT with a cycle length of > 220 ms and 4 had a negative study on study 2. Two of these last patients had a revascularization. The only one patient with inducible VF with 3 extrastimuli had still inducible VF with one extrastimulus.

Twenty-five patients had a negative PVS on study 1; 7 of them had inducible VT on study 2, 4 patients had inducible VFl and in 14 patients PVS remained negative.

### PVS reproducibility

Total reproducibility of programmed stimulation was 70 %. The reproducibility was 100 % when sustained monomorphic VT was initially induced with the induction of a VT or a VFl at second study. When VFl or VF was initially induced, VT or VFl/VF remained inducible in 71 % of patients. When the first study was negative, it remained negative in only 56 % of patients.

### Factors of changes of PVS

In patients with initially inducible VT, there was a trend for an increase of QRS duration from 128±25 ms to 138±25 and a trend for a decrease of LVEF (from 34±11 % to 31±12 %) (Table 3).

In patients with initially inducible VFl or VF (Table 4), QRS duration increased in all patients. The differences were significant in patients with still inducible VT or VFl/VF and were not significant in those with negative study. LVEF decreased significantly in patients with still inducible VT or VFl/VF and there was a trend for a decrease in patients with negative study. However the main decrease of LVEF was noted in patients with inducible VT at second study. Surprisingly, all patients but two with inducible ventricular flutter by 2 extrastimuli at the first study had a negative PVS at second study. Four patients with inducible ventricular flutter by 3 extrastimuli at the first study had inducible VT at the second PVS.

The changes of the results of PVS on study 2 in patients with initially negative PVS (Table 5) were not correlated with a prolongation of QRS duration (Table 3), but with a significant decrease of LVEF noted in patients with inducible VT at second study. In patients with still negative PVS, LVEF did not change. QRS duration was longer at the first study in patients who had inducible VT or VFL/VF at second study than in those who had a second negative study.

The induction of VT in patients with initially negative PVS and in patients with inducible VFl/VF was associated with a significant decrease of LVEF from 41±8 to 35±7 % (p 0.05) and a stability of QRS duration (130±18 vs 142±29) (p 0.5),  A negative PVS at 2nd in patients with initially negative study and with induced VFl//VF was associated with a stability of LVEF (39±16 vs 38±14 %) and QRS duration (106±18  vs 116±18 ms (NS) and these patients had a smaller QRS duration at the first PVS than patients who will have inducible VT at second PVS (106±18 vs 130±18 ms) (p 0.01).

Revascularization was associated with a negative PVS in 2 patients with initially inducible VFl and did not change the induction of VT. Dizziness at second study did not change the results of second study when first study was negative. Of 4 patients with dizziness, 2 patients had still negative study and 2 patients had inducible VT. ICD was implanted in all patients with induced VT and ventricular flutter after the second PVS.

## Discussion

The study indicates that after MI, VT remains inducible several years later from 3 to 14 years. In patients without inducible VT, the second study remains negative in only 56 % of patients and these changes could explain that ICD was useful in some patients with old MI, in spite of initial negativity of PVS. In patients with initially inducible ventricular flutter or fibrillation, the induction of a monomorphic VT was associated with a decrease of LVEF. The decrease of LVEF was also the main factor associated with the induction of a VT at the second study when the patient had a negative initial study. The negativation of the induction of a ventricular flutter could be related to a revascularization. The number of extrastimuli required to induce the ventricular flutter did not explain the changes of the second PVS.

The study confirms:

- The significance of monomorphic sustained VT induction after myocardial infarction.
- The necessity to indicate a revascularization which was not systematic in the past and to look for the coronary status if a ventricular flutter or fibrillation is induced.
- The necessity to follow our patients and to be careful if initial QRS duration is prolonged (> 119 ms) and when LVEF decreased during the time.

Progression of coronary stenosis, left ventricular remodelling, change of medications and revascularization are causes likely to modify the results of PVS. Previous studies had demonstrated generally the high reproducibility of VT induction from 76 % to 96 % during short periods of time from several hours [17,18] or days [21] up to several months [22,23] or up to 4 years [24]. In last study of our group, we reported an increase of QRS duration which does not remain significant several years later. Non-reproducibility of the PVS has been shown in patients with non sustained VT [25-28].

In the present study, we confirm the long-term high reproducibility of sustained VT induction. However, the mode of VT induction was reported to vary from one to another [19-21] and variations of VT morphology were also reported in patients with MI [22,29], but some of these VTs are reflecting the same reentrant circuit with different exit.

Most of these previous studies differed from our study because they included only patients with spontaneous ventricular arrhythmias [17-23]. None of our patients had spontaneous VT.  Our patients without spontaneous VT but with inducible monomorphic VT in 11 cases or VFl, VF in 14 cases were generally recruited initially before the results of MADIT I study [31] or MUSTT study [11] and did not receive ICD even when LVEF was less than 35 %; they did not receive antiarrhythmic drugs because a study of our group did not find change of prognosis with electrophysiologically-guided antiarrhythmic therapy [14]. In patients without inducible VT at the first study, sustained VT or VF can be induced in 44 % of them, several years later, because of change of the arrhythmogenic substrate [30]. Some studies reported a progressive dilatation of left ventricle beyond one year after acute MI [32]. In our study, the initially increasing QRS duration and then, the significant decrease of LVEF was associated with the induction of a VT in patients with initially negative study.  Initial increasing QRS duration (> 119 ms) and then decreasing LVEF in patients with initially induced VFl/VF, which is an arrhythmia of debatable significance [33], was associated with still induction of VT or VF.  At opposite the number of extrastimuli required to induce the first arrhythmia was not a cause for a change of the results of second PVS.

### Limitations of the study

The repetition of PVS was not randomly performed. Clinical conditions were the reasons of a second study, as occurrence of non sustained ventricular arrhythmias or dizziness. Elimination of class II-IV angina or heart failure excluded an important category from this study. The induced arrhythmia could have changed in these patients. Some treatments as statin use or the dosage of ACE inhibitors have changed during this long period of follow-up. The lack of significance of the changes of QRS duration and LVEF in some subgroups of our study, who had modifications of their PVS, is probably related to the small number of patients.

 In conclusion, when the classical stimulation protocol is used, the long-term reproducibility of the induced sustained VT is high in patients without spontaneous VT but with inducible sustained VT at the first study. These patients could be considered as candidates for the prophylactic therapies reported in MADIT study, even several years after acute MI when spontaneous LVEF is less than 35 % and for therapeutic indications of ICD in the case of dizziness or syncope. In patients without induced VT at the first study, there are possible changes of PVS through the life which are related to initial QRS duration and modifications of LVEF. Patients with initially long QRS duration (> 119 ms) and patients who developed decreased LVEF are more at risk to have inducible monomorphic VT at second study, than patients without initially wide QRS and without decrease of LVEF. These findings could also explain the low sensitivity of PVS in MADIT II study.

## Figures and Tables

**Table 1:**
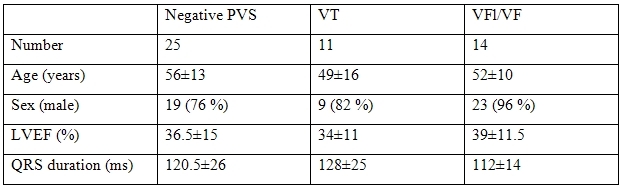
Data of the population after acute MI

**Table 2:**
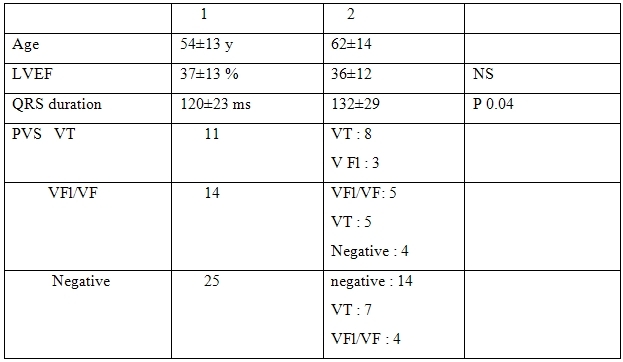
General modifications of clinical and electrophysiological data through 7.5 years

**Table 3:**
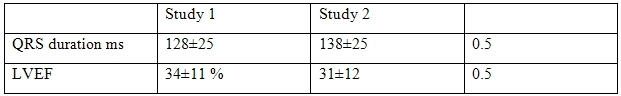
Correlations between the changes of the results of PVS and the data of non-invasive studies in patients with initially inducible VT

**Table 4:**
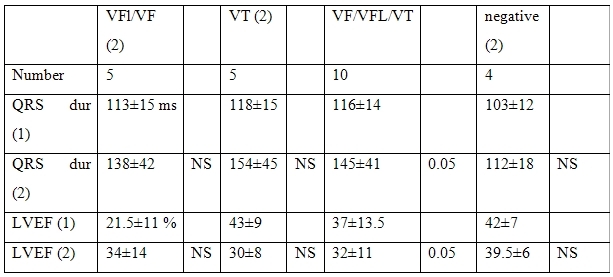
Correlations between the changes of the results of PVS and the data of non-invasive studies in patients with initially inducible VF or/VF (QRS dur: QRS duration)

**Table 5:**
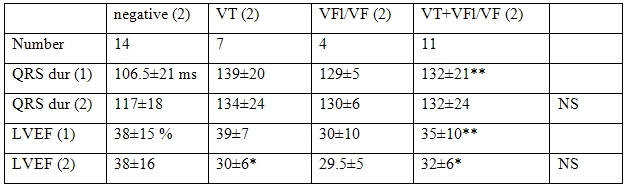
Correlations between the changes of the results of PVS at study 2 (2) and the data of non-invasive studies in patients with initially negative PVS

The statistical analysis compared the values at study 1 and 2 between patients with negative study and patients with inducible VT or VFl/VF at study 2 (** p < 0.01)
